# Supplementary material for: Using the past to estimate sensory uncertainty
Source: eLife. 2020 Dec 15;9:e54172. doi: 10.7554/eLife.54172 (PMC7806269; doi:10.7554/eLife.54172)
Supplement: Supplementary file 1. [file elife-54172-supp1.docx]

**Supplementary file 1**

| **Supplementary file 1-Table 1.** Minimum number of trials per bin across participants for all four sequences. | | | | | | | | | | | | | | | | | | | | |
| --- | --- | --- | --- | --- | --- | --- | --- | --- | --- | --- | --- | --- | --- | --- | --- | --- | --- | --- | --- | --- |
|  | Bin number | | | | | | | | | | | | | | | | | | | |
|  | 1 | 2 | 3 | 4 | 5 | 6 | 7 | 8 | 9 | 10 | 11 | 12 | 13 | 14 | 15 | 16 | 17 | 18 | 19 | 20 |
| Sin | 61 | 54 | 60 | 55 | 52 | 54 | 66 | 55 | 54 | 54 | 66 | 54 | 62 | 53 | 56 | 53 | 61 | 52 | 62 | 52 |
| RW1 | 52 | 55 | 56 | 56 | 56 | 54 | 54 | 57 | 56 | 55 | 57 | 56 | 62 | 59 | 58 | 56 | 57 | 56 | 56 | 55 |
| RW2 | 75 | 88 | 77 | 84 | 77 | 90 | 77 | 88 | 71 | 85 | 74 | 87 | 75 | 84 | 78 | 85 | 80 | 85 | 71 | 86 |
| Sinusoidal jumps | 44 | 47 | 48 | 48 | 45 | 50 | 50 | 49 | 49 | 50 | 51 | 50 | 50 | 48 | 49 | - | - | - | - | - |
| **Note:** The sinusoidal jump sequence had less trials per bin because the sequence was analyzed in three pooled sub-types of up- and down-jumps. | | | | | | | | | | | | | | | | | | | | |

| **Supplementary file 1-Table 2.** The influence of the visual location on the previous trial on A) the perceived sound location of the current trial and its correlation with visual noise in B) the current and C) the previous trial. | | | | |
| --- | --- | --- | --- | --- |
|  | Mean across participants ± SEM | t | df | p |
| **A** | ß_Vprevious_ |  |  |  |
| Sinusoidal | -0.017±0.007 | -2.545 | 24 | 0.018 |
| RW1 | -0.024±0.008 | -2.913 | 32 | 0.006 |
| RW2 | -0.009±0.012 | -0.771 | 18 | 0.45 |
| Sinusoidal jumps | 0.019±0.009 | 2.046 | 17 | 0.057 |
| **B** | r(ß_Vprevious,bin_, σ_Vcurrent,bin_) |  |  |  |
| Sinusoidal | -0.082±0.059 | -1.377 | 24 | 0.181 |
| RW1 | -0.003±0.056 | -0.052 | 32 | 0.959 |
| RW2 | -0.069±0.067 | -1.04 | 18 | 0.312 |
| Sinusoidal jumps | 0.155±0.061 | 2.567 | 17 | 0.020 |
| **C** | r(ß_Vprevious,bin_, σ_Vprevious,bin_) |  |  |  |
| Sinusoidal | -0.076±0.052 | -1.454 | 24 | 0.159 |
| RW1 | 0.004±0.057 | 0.071 | 32 | 0.944 |
| RW2 | 0.009±0.062 | 0.149 | 18 | 0.883 |
| Sinusoidal jumps | 0.080±0.055 | 1.463 | 17 | 0.162 |
| **A** The subject-specific ß_Vprevious_ quantify the influence of the visual location on the previous trial on the perceived sound location of the current trial averaged across all bins. They were entered into a one-sample t-test (against zero) at the group level.  **B** We correlated ß_Vprevious,bin_ with the bin-average standard deviation of the visual cloud of dots in the *current* trial r(ß_Vprevious,bin_, σ_Vcurrent,bin_) over bins within each subject and entered these subject-specific Fisher z-transformed correlation coefficients r(ß_Vprevious,bin_, σ_Vcurrent,bin_) into one-sample t-tests (tested against zero) at the group level.  **C** We correlated ß_Vprevious,bin_ with the with the bin-average standard deviation of the visual cloud of dots in the *previous* trial r(ß_Vprevious,bin_, σ_Vprevious,bin_) over bins within each subject and entered these subject-specific Fisher z-transformed correlation coefficients r(ß_Vprevious,bin_, σ_Vprevious,bin_) into one-sample t-tests (tested against zero) at the group level.  For A, B, C we report across participants‘ mean (±SEM), t-value, df = degree of freedom and p-value. | | | | |

| **Supplementary file 1-Table 3.** Analyses of the temporal asymmetry of the relative auditory weights across the four sequences of visual noise using repeated measures ANOVAs with the factors sequence part (1^st^ vs. flipped 2^nd^ half), bin and jump position (only for the sinusoidal sequences with intermittent jumps) when controlling for the location of the cloud of dots in the previous trial. | | | | | | |
| --- | --- | --- | --- | --- | --- | --- |
|  | Effect | F | df1 | df2 | p | Partial η^2^ |
| Sinusoid | Part | 9.483 | 1 | 24 | 0.005 | 0.283 |
|  | Bin | 86.285 | 2.924 | 70.176 | <0.001 | 0.782 |
|  | PartXBin | 2.165 | 2.633 | 63.190 | 0.109 | 0.083 |
| RW1 | Part | 14.206 | 1 | 32 | <0.001 | 0.307 |
|  | Bin | 78.099 | 5.003 | 160.081 | <0.001 | 0.709 |
|  | PartXBin | 1.454 | 5.055 | 161.755 | 0.207 | 0.043 |
| RW2 | Part | 3.502 | 1 | 18 | 0.078 | 0.163 |
|  | Bin | 61.008 | 3.276 | 58.968 | <0.001 | 0.772 |
|  | PartXBin | 3.450 | 4.655 | 83.799 | 0.008 | 0.161 |
| Sinusoid with intermittent jumps | Jump | 6.493 | 1.034 | 17.586 | 0.020 | 0.276 |
|  | Part | 9.295 | 1 | 17 | 0.007 | 0.353 |
|  | Bin | 64.629 | 2.274 | 38.662 | <0.001 | 0.792 |
|  | JumpXPart | 0.100 | 1.020 | 17.336 | 0.760 | 0.006 |
|  | JumpXBin | 13.259 | 4.043 | 68.732 | <0.001 | 0.438 |
|  | PartXBin | 0.769 | 4.311 | 73.283 | 0.558 | 0.043 |
|  | JumpXPartXBin | 2.180 | 4.820 | 81.945 | 0.066 | 0.114 |
| **Note:** The relative auditory weights were computed in a regression model that included the location of the cloud of dots in the previous trial as a nuisance covariate. The factor bin comprised 9 levels in the first three and 7 levels in the fourth sequence. In this sequence, the factor Jump comprised three levels. If Mauchly tests indicated significant deviations from sphericity (p < 0.05), we report Greenhouse-Geisser corrected degrees of freedom and p values. | | | | | | |

| **Supplementary file 1-Table 4.** The effect of the visual STD in the current bin and the difference in STD between the current and the previous bin on the relative auditory weights when controlling for the location of the cloud of dots in the previous trial. | | | | | |
| --- | --- | --- | --- | --- | --- |
|  |  | t | df | p | Cohen’s d |
| Sinusoid | ß_σV_ | 15.188 | 24 | <0.001 | 3.038 |
|  | ß_ΔσV_ | -3.444 | 24 | 0.002 | -0.689 |
| RW1 | ß_σV_ | 16.221 | 32 | <0.001 | 2.824 |
|  | ß_ΔσV_ | -2.815 | 32 | 0.008 | -0.490 |
| RW2 | ß_σV_ | 13.017 | 18 | <0.001 | 2.986 |
|  | ß_ΔσV_ | -2.530 | 18 | 0.021 | -0.580 |
| Sinusoid with intermittent jumps | ß_σV_ | 11.593 | 17 | <0.001 | 2.733 |
|  | ß_ΔσV_ | -4.915 | 17 | <0.001 | -1.159 |
| **Note**: The relative auditory weights w_A,bin_ were computed in a first regression model that included the location of the cloud of dots in the previous trial as a nuisance covariate. Then we computed a second regression model to assess whether w_A,bin_ was predicted not only by the visual cloud’s STD of the current, but also of the previous bin using the following regression model: w_A,bin_= σ_V,bin_ * ß_σV_ + (σ_V,bin_ – σ_V,bin-1_)* ß_ΔσV_  + ß_const_ + e_bin_ with w_A,bin_= relative auditory weight in bin b; σ_V,bin_ = mean visual STD in current bin b or previous bin b-1; ß_const_ = constant term; e_bin_ = error term. To allow for generalization to the population level, the parameter estimates (ß_σV,_ ß_ΔσV_) for each subject were entered into two-sided one-sample t-tests at the between-subject random-effects level. | | | | | |

| **Supplementary file 1-Table 5**. Nested model comparison of linear mixed-effects models predicting the relative auditory weights w_A,bin_ by the visual STD in the current bin (reduced model) and additionally the difference in STD between the current and the previous bin (full model). | | | |
| --- | --- | --- | --- |
|  | LLRT | p | BIC diff |
| Sinusoidal | 33.584 | <0.001 | -27.369 |
| RW1 | 26.074 | <0.001 | -19.582 |
| RW2 | 39.049 | <0.001 | -33.109 |
| Sinusoidal jump | 21.205 | <0.001 | -14.508 |
| The full and reduced linear mixed-effects models were fitted using maximum likelihood estimation and statistically compared using a likelihood ratio test (LLRT). The difference in model fit is indicated by the difference in Bayesian information criterion (BIC). Negative BIC values indicate greater evidence for the full model relative to the reduced model. | | | |

| **Supplementary file 1-Table 6.** Observers’ and models’ relative auditory weights before versus after the up- and down jumps and their deviations from model predictions. | | | | | | | |
| --- | --- | --- | --- | --- | --- | --- | --- |
|  |  | Up-jump | | | Down-jump | | |
|  |  | t | df | p | t | df | p |
| w_A_ | Behavior | -3.430 | 15 | 0.004 | 1.649 | 15 | 0.12 |
|  | Instantaneous learner | -9.950 | 15 | <0.001 | 7.164 | 15 | <0.001 |
|  | Bayesian learner | -9.774 | 16 | <0.001 | 4.947 | 14 | <0.001 |
|  | Exponential learner | -8.542 | 17 | <0.001 | 5.872 | 15 | <0.001 |
| Squared error of models | Instantaneous learner | 1.599 | 13 | 0.134 | 2.376 | 13 | 0.034 |
|  | Bayesian learner | 1.235 | 14 | 0.237 | 2.350 | 12 | 0.037 |
|  | Exponential learner | 1.085 | 15 | 0.295 | 2.335 | 13 | 0.036 |
| **Note:** We computed w_A_ selectively for sampling time points at 0.1 s before and 0.1 s after the jumps (pooled over all jump types). w_A_ was compared before and after jumps in paired t-tests. We computed the squared error (SE) as (w_A,behavior_ – w_A,model_)^2^ , i.e. the squared difference between the w_A_ based on observers’ behavior and the predictions of the instantaneous, the exponential and the Bayesian learner separately for sampling time points at 0.1 s before and 0.1 s after the jumps (pooled over all jump types). Because time points included only few trials in some participants, individual w_A_ values that were smaller or larger than three times the scaled median absolute deviation were excluded from the analysis. We compared the SEs before versus after the jumps at the group level using two-sided paired t tests. | | | | | | | |

| **Supplementary file 1-Table 7.** Percentage of bias and variability of model parameters from model recovery. | | | | | | | |
| --- | --- | --- | --- | --- | --- | --- | --- |
| Model | Para-meter | Bias | | | Variability | | |
|  |  | Q1 | Median | Q3 | Q1 | Median | Q3 |
| Instantaneous learner | σ_A_ | -5.36 | -0.53 | 1.62 | 2.00 | 2.13 | 2.95 |
|  | P_common_ | -4.23 | -1.49 | 0.15 | 2.12 | 2.34 | 3.80 |
|  | σ_0_ | -3.12 | 1.81 | 5.01 | 2.62 | 3.42 | 9.45 |
| Bayesian learner | σ_A_ | -6.70 | -0.17 | 2.15 | 2.36 | 3.22 | 5.52 |
|  | κ | -9.51 | 9.71 | 68.16 | 14.71 | 32.09 | 85.62 |
|  | P_common_ | -3.86 | -1.13 | 7.00 | 1.62 | 2.07 | 3.84 |
|  | σ_0_ | -2.26 | 2.68 | 4.97 | 3.00 | 4.45 | 6.79 |
| Exponential learner | σ_A_ | -6.67 | -1.57 | 1.76 | 2.96 | 3.09 | 3.12 |
|  | γ | -5.43 | 4.43 | 8.02 | 6.17 | 7.11 | 12.69 |
|  | P_common_ | -4.92 | -2.23 | 0.18 | 2.63 | 3.28 | 4.47 |
|  | σ_0_ | -3.5 | 2.13 | 5.86 | 2.68 | 4.24 | 6.95 |
| Note: The bias is computed by the percentage deviation from the true generating value. Variability is computed by the percentage absolute deviation from the true generating value. Q1 = first quartile; Q3 = third quartile | | | | | | | |
