# Supplementary material for: Using the past to estimate sensory uncertainty
Source: eLife. 2020 Dec 15;9:e54172. doi: 10.7554/eLife.54172 (PMC7806269; doi:10.7554/eLife.54172)
Supplement: Transparent reporting form [file elife-54172-transrepform.docx]

***eLife’s* transparent reporting form**

We encourage authors to provide detailed information *within their submission* to facilitate the interpretation and replication of experiments. Authors can upload supporting documentation to indicate the use of appropriate reporting guidelines for health-related research (see [EQUATOR Network](http://www.equator-network.org/%20)), life science research (see the [BioSharing Information Resource](https://biosharing.org/)), or the [ARRIVE guidelines](http://www.plosbiology.org/article/info:doi/10.1371/journal.pbio.1000412) for reporting work involving animal research. Where applicable, authors should refer to any relevant reporting standards documents in this form.

If you have any questions, please consult our Journal Policies and/or contact us: [editorial@elifesciences.org](mailto:editorial@elifesciences.org).

**Sample-size estimation**

- You should state whether an appropriate sample size was computed when the study was being designed
- You should state the statistical method of sample size computation and any required assumptions
- If no explicit power analysis was used, you should describe how you decided what sample (replicate) size (number) to use

Please outline where this information can be found within the submission (e.g., sections or figure legends), or explain why this information doesn’t apply to your submission:

The sample sizes of 20-38 participants were based on pilot experiments, which showed robust effects of past visual noise on the weighting of audiovisual spatial signals (i.e., the effect was individually significant in 6/6 pilot participants for the sinusoidal sequence). Thus, we decided to collect ≥ 20 participants to get sufficient power at the group level, without formal sample size computation. This information can be found in the Methods section – Subject numbers and inclusion criteria.

**Replicates**

- You should report how often each experiment was performed
- You should include a definition of biological versus technical replication
- The data obtained should be provided and sufficient information should be provided to indicate the number of independent biological and/or technical replicates
- If you encountered any outliers, you should describe how these were handled
- Criteria for exclusion/inclusion of data should be clearly stated
- High-throughput sequence data should be uploaded before submission, with a private link for reviewers provided (these are available from both GEO and ArrayExpress)

Please outline where this information can be found within the submission (e.g., sections or figure legends), or explain why this information doesn’t apply to your submission:

Each of the four experiments replicated the effect of past visual noise on the weighting of audiovisual stimuli. In the Sin, RW1 and RW 2 sequence experiments, participants partially participated in multiple experiments (i.e. a technical replication) while others participated in one experiment so that the samples were not identical (i.e. biological replicates). In the sinusoidal jump sequence experiment, an independent sample participated (i.e., a biological replicate). Each of the four experiments was performed once. In the Methods section – Subject numbers and inclusion criteria, we report the information in more detail. Inclusion/exclusion criteria are also reported in this section. We did not exclude outliers (except in additional analyses in Figure 4-figure supplement 2 and and Supplementary file 1-Table 5).

**Statistical reporting**

- Statistical analysis methods should be described and justified
- Raw data should be presented in figures whenever informative to do so (typically when N per group is less than 10)
- For each experiment, you should identify the statistical tests used, exact values of N, definitions of center, methods of multiple test correction, and dispersion and precision measures (e.g., mean, median, SD, SEM, confidence intervals; and, for the major substantive results, a measure of effect size (e.g., Pearson's r, Cohen's d)
- Report exact p-values wherever possible alongside the summary statistics and 95% confidence intervals. These should be reported for all key questions and not only when the p-value is less than 0.05.

Please outline where this information can be found within the submission (e.g., sections or figure legends), or explain why this information doesn’t apply to your submission:

We describe the statistical analyses methods in detail in the Methods–Data analysis and the computational modeling analyses in Methods– Computational Models and Methods–Model estimation and comparison. In the Results section and Table 1, we report statistical tests, test statistics, p values and effect sizes for each experiment. In Fig. 2-4 we report across-subject means and in Fig. 2 also SEMs. N is reported in the legend of Fig. 2 and Fig. 4. We do not report raw data because this would heavily clutter our figures given our N and the complexity of the figures.

(For large datasets, or papers with a very large number of statistical tests, you may upload a single table file with tests, Ns, etc., with reference to sections in the manuscript.)

**Group allocation**

- Indicate how samples were allocated into experimental groups (in the case of clinical studies, please specify allocation to treatment method); if randomization was used, please also state if restricted randomization was applied
- Indicate if masking was used during group allocation, data collection and/or data analysis

Please outline where this information can be found within the submission (e.g., sections or figure legends), or explain why this information doesn’t apply to your submission:

Because we used a within-subjects design with single groups of healthy participants across the four sequences, we did not allocate experimental groups.

**Additional data files (“source data”)**

- We encourage you to upload relevant additional data files, such as numerical data that are represented as a graph in a figure, or as a summary table
- Where provided, these should be in the most useful format, and they can be uploaded as “Source data” files linked to a main figure or table
- Include model definition files including the full list of parameters used
- Include code used for data analysis (e.g., R, MatLab)
- Avoid stating that data files are “available upon request”

Please indicate the figures or tables for which source data files have been provided:

Raw behavioral data and model predictions as well as the code for computational modelling and analyses scripts are available in an OSF repository: <https://osf.io/gt4jb/> . All parameters of the computational models are reported in Table 2.
